# The non-bacterial oncobiome: the role of the mycobiome and virome in tumor plasticity

**DOI:** 10.1186/s43046-026-00370-x

**Published:** 2026-05-28

**Authors:** Isaac Johnson Ajeh, Ozhe Sunday Isaac Ikukpla’si

**Affiliations:** 1 National Institute for Pharmaceutical Research and Development (NIPRD), Abuja, Nigeria; 2 Federal University Teaching Hospital, Lafia, Nigeria

**Keywords:** Epithelial-mesenchymal transition, Lineage plasticity, Pattern recognition receptor, Phenotypic switching, Tumor microenvironment

## Abstract

Tumor plasticity, the capacity of malignant cells to undergo reversible phenotypic switching, is a fundamental driver of lineage diversion and therapeutic resistance. While the bacterial microbiome is a recognized modulator of the tumor microenvironment (TME), the non-bacterial oncobiome, comprising the mycobiome (fungi) and virome (viruses), represents a critical but under-explored frontier in cellular adaptability. This review synthesizes current evidence regarding the mechanistic contributions of fungal and viral constituents to tumor plasticity and characterizes the molecular cross-talk that facilitates host cell reprogramming. We conducted a structured narrative synthesis of literature indexed in PubMed, Scopus, and Web of Science (2020–2026), focusing on high-throughput studies such as ITS sequencing, metagenomics NGS (mNGS), and single-cell network analyses. We specifically evaluated evidence concerning the activation of host pattern recognition receptors and the subsequent transcriptional rewiring of lineage-defining markers. Emerging data indicate that fungal dysbiosis, particularly involving Candida and Malassezia species, triggers the Dectin-1/STAT3 signaling axis, a known inducer of epithelial-mesenchymal transition (EMT). Concurrently, the virome, ranging from integrated oncoviruses to reactivated endogenous retroviruses (ERVs), is shown to hijack the Wnt/ β-catenin pathway, enforcing a progenitor-like stemness state. This inter-kingdom synergy promotes an immune-excluded niche, effectively shielding plastic sub-populations from cytotoxic stress and targeted therapies. The non-bacterial oncobiome provides genomic momentum and inflammatory cues necessary to lower the threshold for phenotypic switching. This review highlights that stabilizing the TME ecosystem through ecologically targeted therapy may be a prerequisite for overcoming drug resistance and improving clinical outcomes in refractory cancers.

## Introduction: redefining the oncobiome

For decades, the study of the cancer microbiome (the oncobiome) focused almost exclusively on the bacterial bystanders or instigators. We understood *Helicobacter pylori* as a gastric carcinogen and recognized how gut *Fusobacterium nucleatum* could influence colorectal cancer progression. However, a significant portion of the tumor’s internal ecosystem remained in the dark matter of biological research: the mycobiome (fungi) and the virome (viruses). These non-bacterial residents are far from mere passengers. Emerging research indicates they are active molecular architects of tumor plasticity, the phenomenon where cancer cells shift their phenotypic identity to survive therapy, evade the immune system, and colonize distant organs [1].

While bacteria are the most numerically dominant microbial inhabitants, fungi and viruses possess unique, high-impact molecular toolkits that can reshuffle the TME [2]. Unlike the relatively static host genome, this microbial landscape is dynamic; shifting fungal diversity or viral reactivation can send biochemical signals to neighboring cancer cells. These signals can trigger an identity crisis in the cell, forcing a transition from a stationary, epithelial state to a mobile, mesenchymal state [1]. This phenotypic switching is a primary driver of drug resistance; a cell that can change its locks (receptors) can easily ignore the keys (targeted therapies) designed to kill it [1].

The mycobiome, though comprising less than 1% of the human body’s microbial biomass, has a disproportionately large influence [3]. Species like *Candida albicans*,* Malassezia globosa*, and Aspergillus are found within solid tumors, impacting cancer dynamics [4]. Fungi contribute to plasticity via pattern recognition signaling, where cell wall components like β-glucans and mannans interact with host receptors like Dectin-1, activating pathways like STAT3 that promote cancer cell stemness and survival [4]. Fungi also metabolically reprogram host cells through unique secondary metabolites that alter epigenetic landscapes, re-coding gene expression without changing DNA sequences [2]. This fungal influence can drive cancer progression and treatment resistance.

The tumor virome is a complex, understudied landscape. Classic oncoviruses like human papillomavirus (HPV) and Epstein-Barr virus (EBV) directly insert oncogenes into the host, driving cancer progression [5]. The bacteriophage community, viruses that infect tumor bacteria, can indirectly modulate the immune response by targeting specific bacterial populations [6]. It is hypothesized that reactivated endogenous retroviruses, triggered by TME stress, may participate in the hijacking of host pathways like Wnt/β-catenin. While transcriptomic correlations suggest a link to progenitor-like states, the direct causal requirement of ERV-derived RNAs for EMT induction remains a speculative model requiring further functional validation [5].

**Fig. 1:**
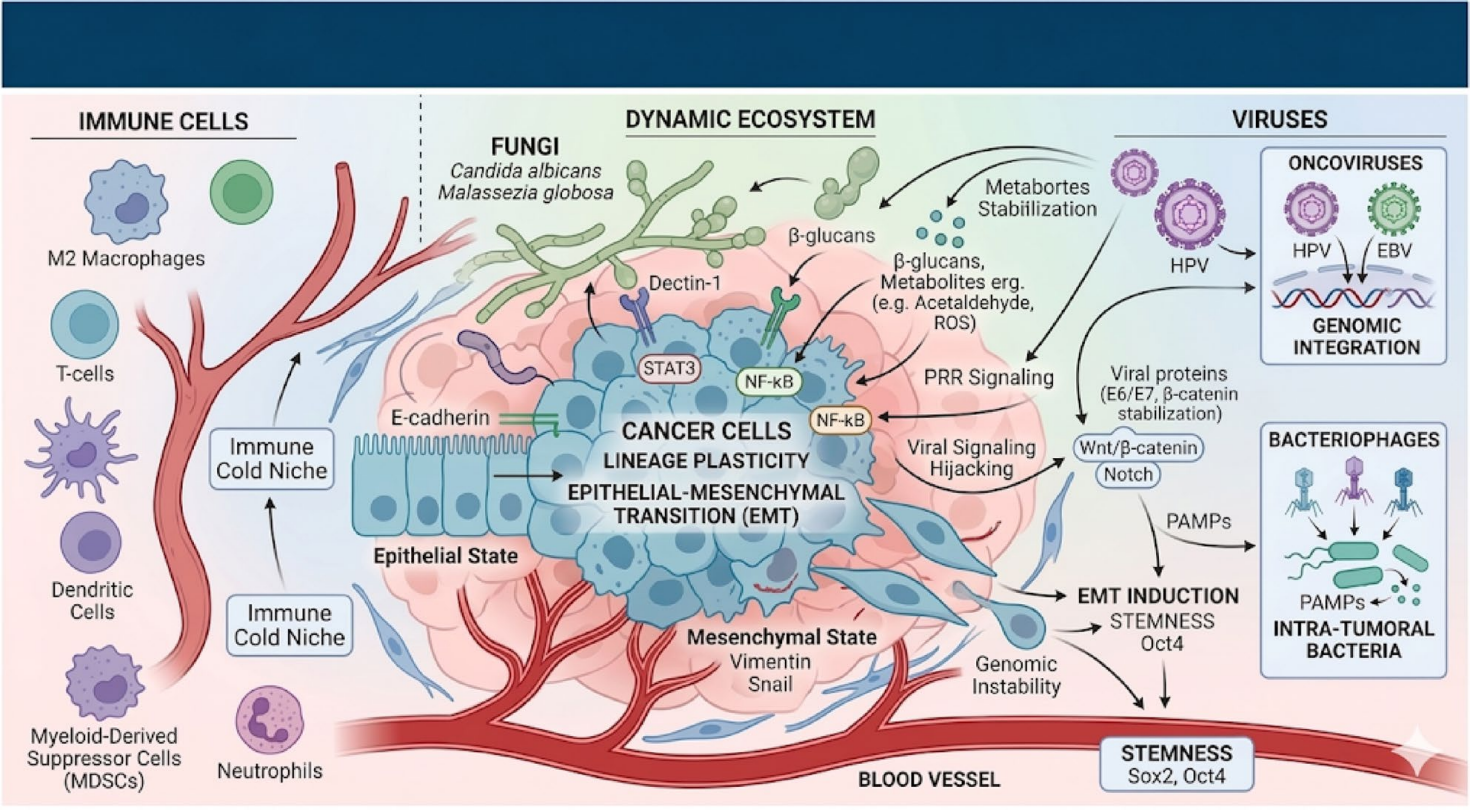
The hidden architecture, showing how fungi (Candida and Malassezia) and viruses (oncoviruses and bacteriophages) are not just present but actively signaling

Critics argue that the altered metabolic state of a plastic tumor (hypoxia, high lactate) simply creates an inviting nich for opportunistic fungi and viruses, rather than these microbes driving the plasticity itself. A topographic overview of the non-bacterial oncobiome, contrasting the traditional bacteria-centric view with the emerging fungal and viral signatures identified via mNGS and ITS sequencing, is presented in Fig. 1.

A cancer cell does not decide to become resistant to chemotherapy in a vacuum; it does so in response to the chemical and biological cues provided by its non-bacterial neighbors [7]. By understanding the non-bacterial oncobiome, we move beyond simple eradication strategies. We enter an era of ecological management, where the goal is not just to kill the cancer cell, but to regulate the ecosystem that allows it to thrive.

Despite the success of targeted therapies, the emergence of lineage plasticity, where cancer cells escape death by switching their cellular identity, remains a primary barrier to a cure. While the bacterial microbiome has been implicated in modulating drug metabolism, the role of the dark oncobiome (fungi and viruses) in actively driving these phenotypic shifts has been largely ignored [8]. There is a critical knowledge gap regarding how non-bacterial signals orchestrate the regulatory rewiring necessary for tumor cells to transition between epithelial, mesenchymal, and stem-like states.

This narrative review is based on a structured synthesis of literature identified through searches of PubMed, Scopus, and Web of Science for articles published between January 2020 and January 2026. The search employed terms including; tumor plasticity, lineage plasticity, mycobiome, tumor virome, Dectin-1, endogenous retroviruses, and epithelial-mesenchymal transition. The inclusion criteria focused on: peer-reviewed primary research and high-impact reviews published in English, studies utilizing high-throughput sequencing (ITS, mNGS) and spatial transcriptomics, and mechanistic evaluation of non-bacterial microbial signalling on host cell identity. The exclusion criteria included studies focusing solely on the bacterial microbiome without inter-kingdom analysis, case reports with low sample sizes, and non-peer-reviewed preprints unless they provided unique mechanistic insights not available elsewhere. A total of 36 primary sources were selected for the final synthesis to ensure a focused evaluation of the non-bacterial drivers of phenotypic switching.

This review aimed to elucidate the emerging role of the non-bacterial oncobiome in driving phenotypic transitions through four thematic areas: landscape analysis identifying fungal and viral signatures linked to tumor plasticity; molecular cross-talk mechanisms triggering EMT via host receptors; transcriptional rewiring of stemness regulators like SOX2; and therapeutic perspectives on modulating the mycobiome/virome to enhance treatment sensitivity.

### Fungal pathogenesis and the induction of lineage plasticity

Fungi are not merely opportunistic colonizers of immunocompromised hosts; they are potent immunomodulators. Recent spatial transcriptomics and high-sensitivity sequencing have confirmed that fungi are not just present in the gut or on mucosal surfaces; they are physically sequestered within the interstitial spaces of solid tumors, including the pancreas, lung, and breast [9].

**Fig. 2:**
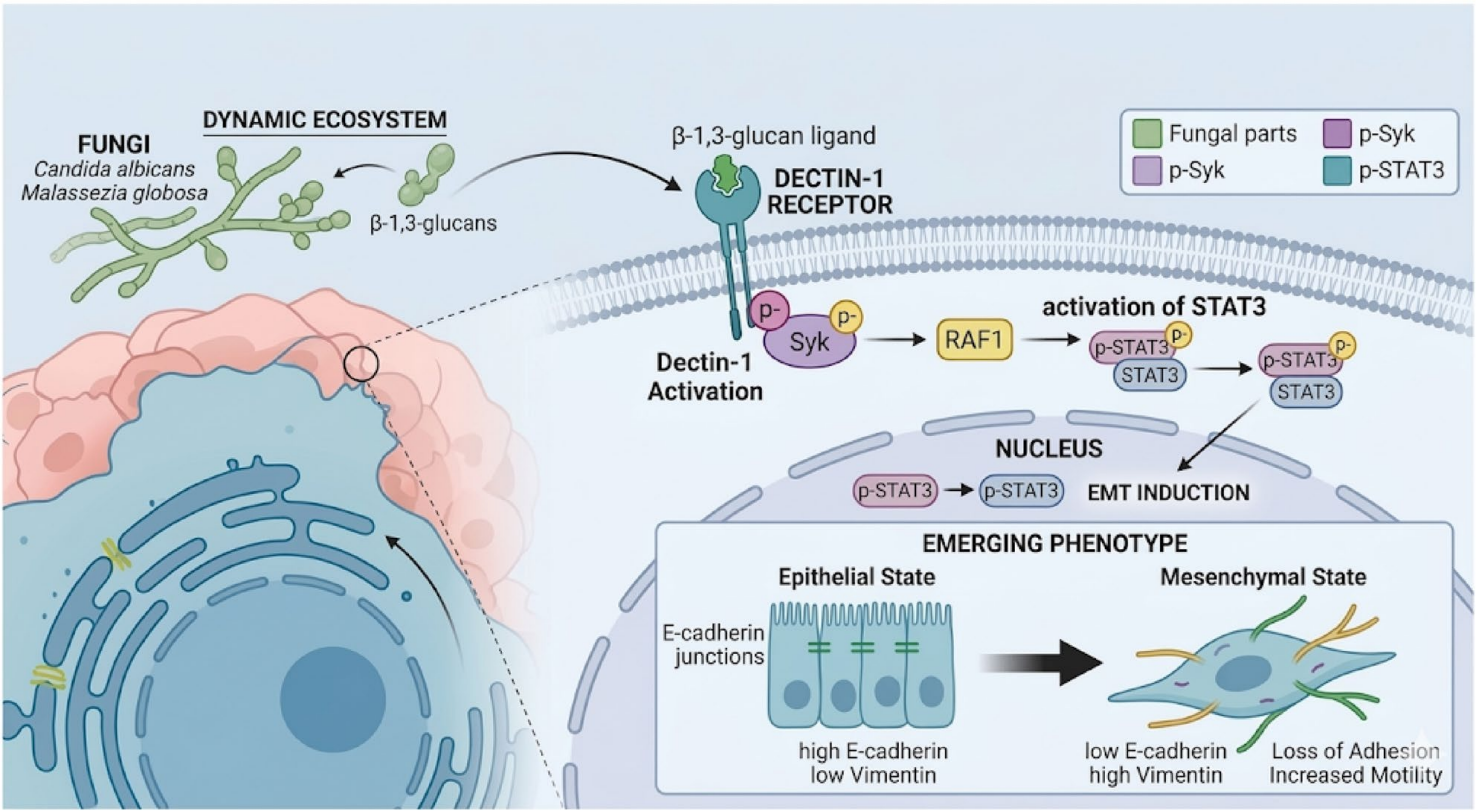
Fungal Pathogenesis. This figure provides a close-up view of the fungal-mesenchymal axis. The emerging mesenchymal phenotype (loss of E-cadherin, high vimentin, spindle shape) is clearly contrasted with the initial epithelial state

Fungal dysbiosis, a shift from a commensal to a pathogenic fungal profile, is often characterized by the overgrowth of specific genera such as Candida, Malassezia, and Aspergillus [8]. Unlike bacteria, which often interact with the host through short-chain fatty acids, fungi possess a complex cell wall rich in pathogen-associated molecular patterns that provide a continuous inflammatory stimulus to the surrounding malignant cells [3]. While Candida is linked to progression in pancreatic ductal adenocarcinoma, some studies suggest that certain fungal cell wall components (like β-glucans) can actually prime the immune system for a better response to immunotherapy in different contexts. Emerging evidence suggests that the activation of the Dectin-1/STAT3 axis by fungal ligands represents a compelling but context-dependent mechanism of plasticity. While high-impact studies in pancreatic ductal adenocarcinoma demonstrate that Malassezia-driven Dectin-1 signaling is a potent inducer of a mesenchymal phenotype, this effect may be tissue-specific. In order contexts, such as certain cutaneous malignancies, Dectin-1 activation has been associated with enhanced anti-tumor immunity rather than EMT. This suggests that the downstream output of the Dectin-1 axis is heavily moderated by the local cytokine milieu and the specific fungal species present, rather than acting as a universal driver of plasticity [10]. The transition from epithelial identity to mesenchymal plasticity is mediated by receptor-ligand interactions detailed in Fig. 2. Notably, the Dectin-1/STAT3 axis (Fig. 2A) highlights how fungal PAMPS initiate the transcriptional rewiring necessary for lineage diversion.

Beyond physical contact, the fungal secretome, the collection of enzymes, toxins, and metabolites secreted by fungi, plays a critical role in metabolic cross-talk [11]. Many Candida species produce high levels of acetaldehyde and reactive oxygen species (ROS) [12]. Chronic exposure to ROS induces oxidative stress, which is a potent trigger for genomic instability and the activation of stress-response pathways (like Nrf2) that favor a stem-like, resistant state [12]. The cytolytic peptide toxin does more than damage membranes; it activates the MAPK/ERK pathway, a central node in cell proliferation and survival that is frequently implicated in resistance to targeted therapies [13].

A landmark observation in mycobiome research is the role of *Malassezia globosa* in pancreatic ductal adenocarcinoma [14]. Research has shown that fungi can migrate from the gut lumen into the pancreatic duct. Once inside the tumor, Malassezia triggers the complement cascade (C3) [15]. The resulting C3a-C3aR signaling on tumor cells directly promotes proliferation and cellular remodeling, providing a clear link between a specific fungal inhabitant and the acceleration of malignancy [15].

The mycobiome contributes to tumor plasticity not through a single mutation, but through a sustained environmental pressure. By maintaining a state of chronic, low-grade inflammation and hijacking host developmental pathways like STAT3 and MAPK, fungi lower the threshold for cancer cells to undergo phenotypic switching [16]. Understanding this fungal-mesenchymal axis is essential for developing adjunct therapies that target the invisible drivers of treatment failure.

### Viral integration and the hijacking of host lineage

While the mycobiome operates largely through external receptor signaling and chronic inflammation, the tumor virome exerts its influence through a more intimate mechanism: the direct manipulation of the host’s genetic and transcriptional machinery [17]. The virome is not a monolithic entity; it is a multi-layered ecosystem consisting of oncogenic viruses, bacteriophages, and ancient endogenous retroelements [17]. The tumor virome comprises distinct viral categories driving plasticity. The effect of viral integration is highly dependent on the site of integration; not all integration events drive stemness and may lead to dead-end mutations that do not confer a survival advantage. Exogenous oncoviruses (HPV-16/18, EBV) represent the most mature evidence base due to proven genomic integration, and it is well-established that HPV E6/E7 oncoproteins degrade tumor suppressors like p53 and Rb, directly driving genomic instability and phenotypic diversion [18]. Bacteriophages shape the bacterial landscape, lysing beneficial bacteria and collapsing immune barriers, enabling phenotypic switching [18]. Endogenous retroviruses, ancient viral remnants (~ 8% of human DNA), can reactivate in the stressed TME, causing transcriptional noise and genomic instability [19].

**Fig. 3:**
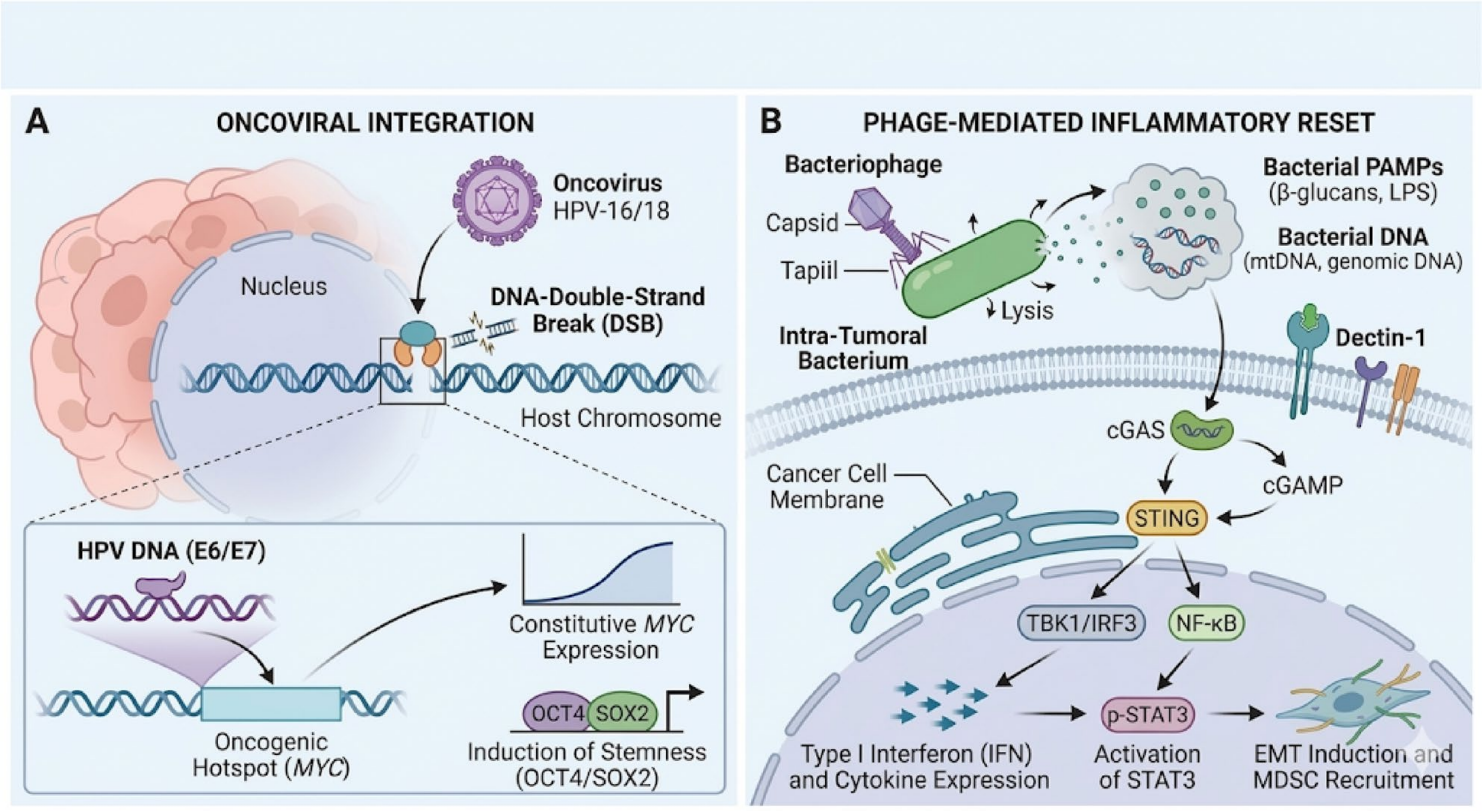
The Virome and Genomic Instability. **A** depicts oncoviral DNA integration, showing HPV DNA (E6/E7) at an oncogenic hotspot (MYC) within the host chromosome. **B** depicts phage-mediated inflammatory reset, illustrating how bacteriophages lyse intra-tumor bacteria to release PAMPs and DNA

One of the most potent drivers of lineage plasticity is the physical integration of viral DNA into the host genome [20]. This process often occurs at regions of the genome that are already open and transcriptionally active [20]. When a virus integrates near a proto-oncogene (e.g., MYC), it can put that gene under the control of a powerful viral promoter. This leads to the constitutive expression of factors that prevent the cell from maturing, forcing it into a progenitor-like or stem-cell state [21]. In several characterized oncoviral models, viral proteins have been shown to stabilize β-catenin, a mechanism that mimics embryonic Wnt signalling. However, whether reactivated endogenous retroelements consistently hijack this pathway across diverse tumor types is a compelling but still largely hypothetical model that requires further functional validation [19–21]. In established oncoviral models like HPV-positive head and neck cancers, the stabilization of β-catenin is a well-documented driver of stemness [17]. However, in the context of reactive endogenous retroviruses, the mechanistic link is less deterministic. The impact of endogenous retroviruses expression on Wnt signalling likely depends on the specific site of genomic reactivation and the presence of co-factors in the TME. As depicted in the integration map of Fig. 3, exogenous oncoviruses act as direct genetic modifiers. By comparing integrated viral loads with Wnt-pathway activity (Fig. 3B), we see a clear correlation between viral persistence and the maintenance of a progenitor-like state.

In healthy cells, endogenous retroviruses are kept in a state of epigenetic lockdown through DNA methylation. However, the hypoxia and oxidative stress typical of a tumor can strip away these epigenetic marks [22]. When endogenous retroviruses are awakened, they produce viral-like RNAs and proteins that the cell perceives as a pseudo-infection [22]. This triggers a Type I Interferon (IFN) response. While IFN is usually anti-viral, chronic low-level IFN signaling in cancer has been shown to paradoxically drive cells toward a mesenchymal-like state and increase the expression of immune checkpoint molecules like PD-L1, allowing the plastic tumor to hide from T-cells [23].

The role of bacteriophages in tumor plasticity is an emerging bystander effect. Phages can transfer antibiotic resistance genes or virulence factors between bacteria within the tumor. More importantly, phage-mediated lysis of bacteria releases PAMPs and DNA into the TME [24]. This sudden influx of microbial debris activates the cGAS-STING pathway in cancer cells [24]. While STING is a target for immunotherapy, its chronic activation can contribute to the secretory phenotype of cancer cells, where they release cytokines that promote their own remodeling and invasion [24].

The virome provides the genomic momentum for tumor evolution. By integrating into the host genome, reactivating ancient retroelements, and manipulating the bacterial landscape, viruses ensure that the tumor cell population remains heterogeneous [25]. This heterogeneity is the bedrock of plasticity; it ensures that even if one cell type is killed by therapy, a viral-reprogrammed subpopulation will survive to regenerate the tumor.

### Kingdom cross-talk—synergy and the total oncobiome

**Fig. 4:**
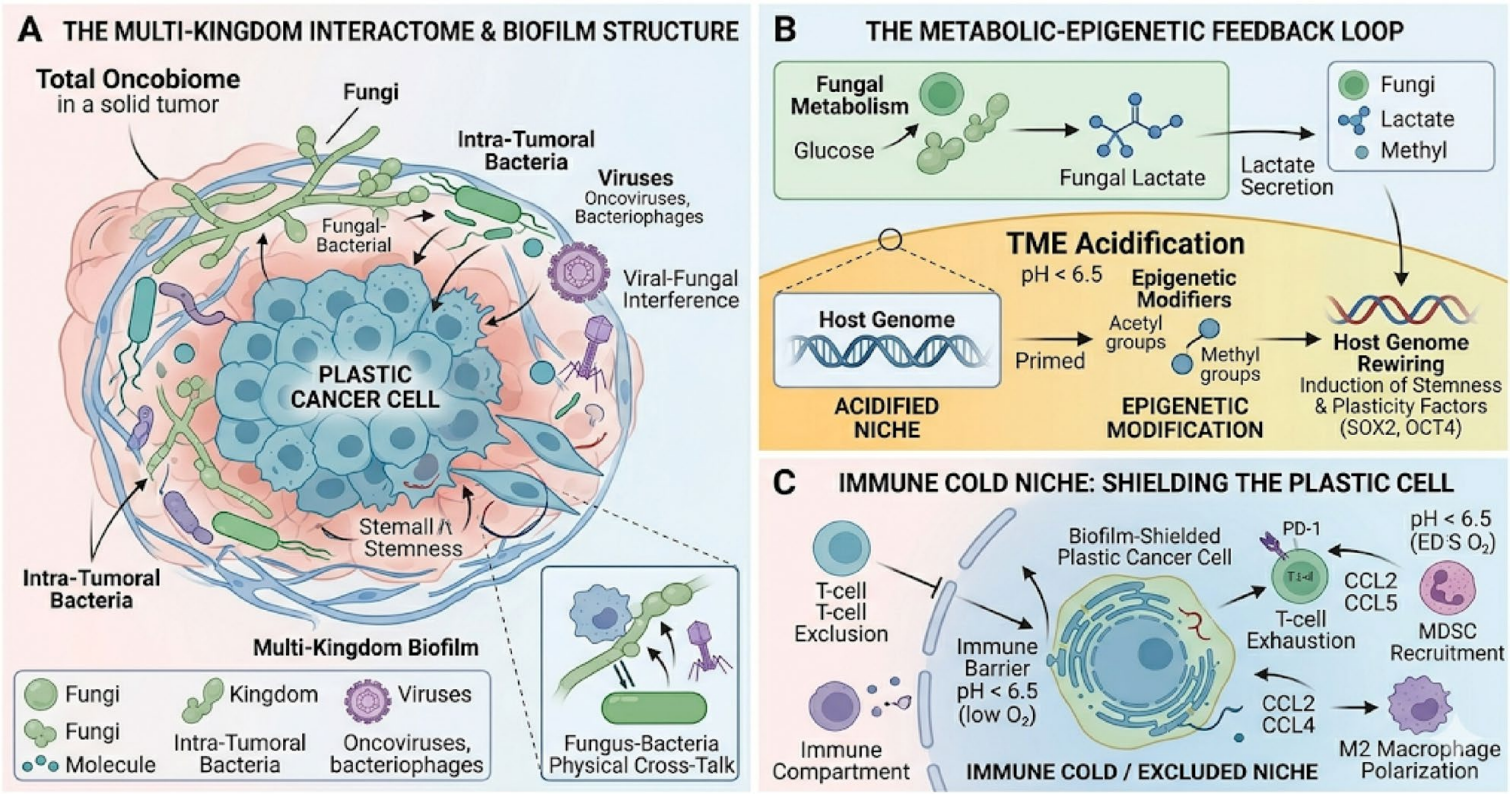
Kingdom Cross-Talk and Synergy. **A** shows the physical interactome, depicting how multi-kingdom biofilms (fungi, bacteria, viruses) shield a centrally located, plastic cancer cell. **B**, details the metabolic loop which causes epigenetic modification (methylation/acetylation) to prime the host genome. **C**, illustrates the functional result: the immune barrier created by the biofilm and acidification

In a typical solid tumor, a fungal hypha may be coated in a biofilm of Pseudomonas, while both are being surveyed by bacteriophages and targeted by host macrophages [26]. This inter-kingdom interactome is hypothesized to create a complex signalling web that potentially lowers the threshold for host cell reprogramming. We propose a model of multi-kingdom synergy wherein the combined inflammatory stimulus from fungal and viral PAMPs may promote an immune-excluded niche (Fig. 4). While initial spatial transcriptomic data indicate correlations between high microbial load and T-cell exhaustion, the direct causal requirement of these microbes for maintaining an immune-cold state is an emerging concept in the field. Rather than acting in isolation, these microbes create a bio-shield-a concept visualized in the multi-layered model in Fig. 4C, where fungal-derived lactic acid and viral microRNAs converge to epigenetically suppress host identity markers. Certain fungi, such as *Candida albicans*, have been observed to provide physical scaffolding for bacteria, which may protect them from the host’s immune cells and systemic antibiotics [27]. In return, bacteria produce metabolites like phenazines that can induce fungal morphogenesis; this yeast to hyphal transition is potentially linked to increased tissue invasion and the release of pro-plasticity cytokines, though the direct causal requirement for plasticity across all tumor types remains to be fully established [27].

While less studied, emerging evidence suggests that certain viral infections may sensitize host cells to fungal ligands, potentially lowering the threshold for Dectin-1 or TLR activation and accelerating the phenotypic shift toward a mesenchymal state [21]. A significant proposed outcome of kingdom cross-talk is the creation of an immune-excluded or cold tumor. Under this synthesized model, when the mycobiome (via β-glucans) and the virome (via chronic Interferon signaling) both stimulate the TME, they may exhaust the local T-cell population, potentially leading to the upregulation of PD-1 and LAG-3 on infiltrating lymphocytes [28]. Fungal-driven inflammation is associated with the recruitment of myeloid-derived suppressor cells, which putatively shield plastic cancer cells from immune surveillance [28]. In this proposed niche, cancer cells may possess the evolutionary breathing room to undergo lineage switching, such as the transformation of lung adenocarcinoma into a small-cell-like neuroendocrine phenotype to escape TKI therapy [28].

Furthermore, both fungi and rapidly dividing plastic cancer cells favor glycolysis (the Warburg effect), leading to an accumulation of lactic acid that may contribute to TME acidification [29]. This acidified environment, combined with viral-encoded microRNAs and fungal secondary metabolites, is hypothesized to act as a potent epigenetic modifier [29]. This putative mechanism suggests a stripping away of identity markers, potentially making the genome more accessible for master regulators of stemness, such as SOX2, NANOG, and OCT4 [29].

Understanding this cross-talk shifts our therapeutic approach. If a tumor’s plasticity is driven by a hypothetical fungal-viral-bacterial axis, targeting only one kingdom (e.g., using only antibiotics) may be counterproductive [30]. Eliminating bacteria could theoretically allow for a fungal bloom, which might inadvertently accelerate EMT through Dectin-1 signaling [30]. Future protocols may require ecologically targeted therapy to stabilize the tumor in treatable state.

Lineage plasticity is conceptualized here not as a solo performance by the cancer cell, but as a potential orchestral arrangement where the mycobiome provides the inflammatory percussion, the virome provides the genomic melody, and the bacteriome sets the tempo to outsmart current pharmacopeia [31].

### Biomarkers, therapeutics, and future frontiers

The shift from a bacterial-centric view to a multi-kingdom total oncobiome model is not merely an academic exercise; it represents a paradigm shift in how we diagnose and treat refractory cancers. If tumor plasticity is an ecologically driven process, then our clinical toolkit must expand to include microbial diagnostics and eco-modulatory interventions.

**Fig. 5:**
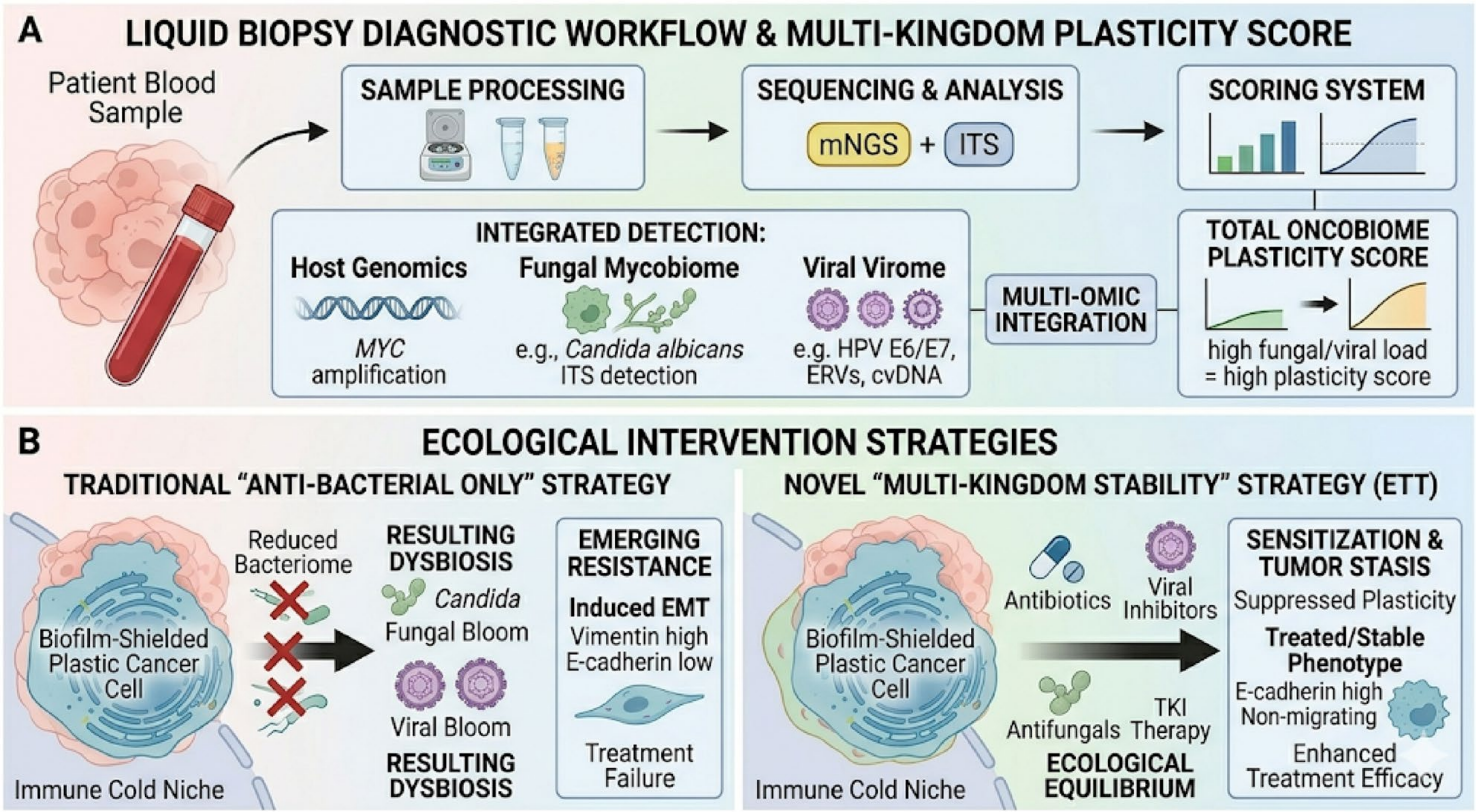
Integration of total oncobiome biomarkers with ecological targeted therapy. **A** illustrates the liquid biopsy diagnostic workflow, demonstrating how processing a simple blood sample can integrate detection of host genetic markers (e.g., MYC), the fungal mycobiome (e.g., Candida ITS), and the viral virome (e.g., HPV E6/E7, ERVs, cvDNA). **B** contrasts two ecological intervention strategies on the biofilm-shielded plastic cancer cell. The left sub-panel, shows how a traditional anti-bacterial-only strategy backfires, reducing bacteria but causing a fungal and viral bloom (the dysbiosis), leading directly to Induced EMT and treatment failure. The right sub-panel illustrates the novel multi-kingdom stability strategy where antibiotics, antifungals, and viral inhibitors are combined with TKI therapy to achieve ecological equilibrium. This combination suppresses plasticity and freezes the orderly, treatable phenotype, leading to sensitization and tumor stasis.

Current oncology relies heavily on host genetics (e.g., EGFR mutations, ALK rearrangements), but a multi-kingdom biomarker panel could offer a more comprehensive approach (Fig. 5A). This panel integrate three key components: the mycobiome score, quantifying the Candida to Saccharomyces ratio as an early EMT warning sign; the viral load and integration map, detecting circulating viral DNA or awakened endogenous retroviruses linked to stemness markers like SOX2; and circulating microbial metabolites signalling tumor plasticity [32, 33]. By combining these biomarkers, clinicians could identify patients at risk of lineage shifts, enabling proactive treatment strategies [33]. The mycobiome score could indicate impending EMT in lung or pancreatic cancers, while the viral load and integration map provide insights into viral-driven plasticity [34]. Circulating microbial metabolites would serve as epigenetic indicators of tumor state changes, allowing for more precise interventions.

While the targeted modulation of the non-bacterial oncobiome represents a compelling frontier, the implementation of ecological targeted therapy (ETT), the simultaneous manipulation of fungi, viruses, and bacteria, remains a highly conceptual framework that is not yet ready for clinical application (Fig. 5B).

The rationale for ETT is currently supported by a small growing body of preclinical evidence (Table 1). For example, recent murine models of pancreatic cancer demonstrated that the pharmacological depletion of intratumoral fungi using amphotericin B or fluconazole significantly slowed tumor progression and synergistically enhanced the efficacy of gemcitabine by reversing Dectin-1-mediated immunosuppression [4, 9]. Similarly, the use of phage therapy to lyse specific pro-tumoral bacteria has shown potential in reducing the protective bacteria niche, theoretically making the tumor more susceptible to conventional chemotherapy [35]. This holistic approach could revolutionize cancer treatment by targeting the tumor ecosystem rather than just the cancer cells themselves. However, the clinical translation of ETT faces significant hurdles and safety concerns: Antifungals often carry heavy metabolic burdens, particularly in patients already undergoing cytotoxic chemotherapy. Broad spectrum antimicrobial interventions risk creating an ecological vacuum. For instance, the elimination of certain bacterial species may lead to a fungal bloom, where opportunistic fungi rapidly colonize the empty niche and inadvertently accelerate EMT through alternative pathways. The unintended disruption of commensal populations in the gut or skin could lead to severe dysbiosis, potentially impairing the host’s overall immune response to the malignancy [36].

**Table 1:** The evidence maturity matrix

Mechanism	Evidence Tier	Primary Basis of Evidence
Exogenous Oncoviral Integration	Tier 1: Established	Validated clinical models; known oncoprotein biochemistry.
Fungal Dectin-1/STAT3 Axis	Tier 2: Emerging	In vivo murine models (e.g., PDAC); high-sensitive clinical correlation sequencing (ITS-seq)
Bacteriophage cGAS-STING Activation	Tier 2: Emerging	Initial in vitro assays; spatial transcriptomic correlations
ERV-driven Lineage Plasticity	Tier 3: Speculative	In silico transcriptomic noise analysis; lacking direct knockout evidence.
Inter-kingdom Biofilm Synergy	Tier 3: Speculative	Hypothesis-driven synthesis; extrapolated from mucosal niche models.

Consequently, ETT should be viewed as a theoretical research direction rather than a current therapeutic recommendation. Future studies must move beyond broad-spectrum depletion toward precision ecological modulation, utilizing tools like engineered phages or small-molecule inhibitors of specific fungal-host receptors (e.g., Dectin-1 antagonists). Such strategies must be rigorously validated in clinical trials to ensure they stabilize tumor plasticity without compromising the patient’s systemic ecological health.

### Critical perspectives: causality, technical limitations, and the road to validation

The integration of the mycobiome and virome into the oncobiome framework provides a compelling explanation for tumor plasticity mechanisms. However, significant biological and technical barriers must be addressed before these models can move from theoretical synthesis to clinical utility.

A major challenge is low-biomass detection and the kitome problem. Fungal and viral constituents often make up less than 1% of the total microbial biomass in a tumor, creating substantial risks for metagenomic next-generation sequencing and ITS analysis [6]. Reagents used for DNA extraction and library preparation frequently contain trace environmental fungal DNA, such as from Aspergillus or Penicillium, which can confound results [21, 25].

Beyond contamination, the field lacks standardized protocols for extracting non-bacterial DNA, leading to high variability between studies [20]. There is also considerable bioinformatic noise [11]. Distinguishing resident, transcriptionally active oncobiome members from transient passenger microbes or environmental contaminants requires rigorous negative controls and specialized pipelines that have not yet been standardized.

Another fundamental issue is reverse causality: do microbial dysbiosis and phenotypic switching drive each other, or does one precede the other? The niche hypothesis suggests that the hypoxic, necrotic, and lactate-rich TME simply creates an acidified space hospitable to opportunistic fungi and viruses. The driver hypothesis argues that microbial ligands like β-glucans and viral oncoproteins such as E6/E7 actively trigger host pathways, including Dectin-1/STAT3 and Wnt/β-catenin, which lower the threshold for EMT and stemness [22].

Evidence increasingly supports a bi-directional feedback loop. Initial microbial signals may prime cellular identity shifts, which in turn further reshape the TME to sustain a complex, multi-kingdom biofilm. This reciprocal interaction complicates efforts to determine what is cause versus effect in tumor progression.

The impact of the oncobiome is also not uniform, and the maturity of evidence varies across microbial groups [13]. Exogenous oncoviruses represent the most established tier, with proven genomic integration and direct manipulation of tumor suppressors through oncoproteins [2]. In contrast, fungal and bacteriophage influences are mostly indirect, acting through secondary inflammatory signaling or broader ecosystem effects.

Conflicting roles further complicate interpretation. While Candida species are often associated with tumor progression, certain fungal cell wall components like -glucans can paradoxically prime the innate immune system and enhance immunotherapy efficacy in specific contexts [5]. Models involving ancient endogenous retroviruses and inter-kingdom synergy, such as fungal-bacterial biofilms, remain largely conjectural and need functional validation.

To advance, the field must shift from associative mapping to causal validation. Standardizing ecological targeted therapy will depend on identifying which microbial signatures are actionable drivers of drug resistance and which are merely bystander markers of advanced disease. Only through rigorous cross-validation between computational network analysis and wet-lab functional assays can we safely modulate the tumor ecosystem to stabilize cancer in a non-plastic, treatable state.

## Conclusion

The history of oncology is a history of chasing a moving target. By incorporating the mycobiome and virome into our understanding of tumor plasticity, we finally see the engine behind that movement. The future of cancer care lies in the transition from search and destroy to stabilize and sensitize. While ETT offers a promising conceptual path to freezing tumor plasticity, it requires extensive validation to navigate the complex risks of systemic microbial imbalance. By managing the microscopic residents that shape the tumor, we can finally strip cancer of its most potent weapon: the ability to change.

## Data Availability

No datasets were generated or analysed during the current study.

## Abbreviations

EMT: Epithelial-Mesenchymal Transition
ERVs: Endogenous Retroviruses
NSCLC: Non-Small Cell Lung Cancer
SCLC: Small Cell Lung Cancer
PDAC: Pancreatic Ductal Adenocarcinoma
TME: Tumor Microenvironment
TKI: Tyrosine Kinase Inhibitor
PRRs: Pattern Recognition Receptors
PAMPs: Pathogen-Associated Molecular Patterns
MDSCs: Myeloid-Derived Suppressor Cells
ROS: Reactive Oxygen Species
IFN: Interferon
cvDNA: Circulating Viral DNA
Dectin-1: Dendritic Cell-Associated C-Type Lectin-1
TLRs: Toll-Like Receptors
STAT3: Signal Transducer and Activator of Transcription 3
NF-kappaB: Nuclear Factor Kappa-Light-Chain-Enhancer of Activated B Cells
Wnt/beta-catenin: Wingless-related Integration Site/Beta-catenin pathway
MAPK/ERK: Mitogen-Activated Protein Kinase/Extracellular Signal-Regulated Kinase
cGAS-STING: Cyclic GMP-AMP Synthase-Stimulator of Interferon Genes
SOX2: SRY-Box Transcription Factor 2
OCT4: Octamer-Binding Transcription Factor 4
NANOG: Nanog Homeobox
PD-1/PD-L1: Programmed Cell Death Protein 1/Ligand 1
LAG-3: Lymphocyte-Activation Gene 3
ITS: Internal Transcribed Spacer (Sequencing for fungi)
mNGS: Metagenomic Next-Generation Sequencing
NGS: Next-Generation Sequencing
ETT: Ecological Targeted Therapy
RAF1: Rapidly Accelerated Fibrosarcoma 1
IOM: Immuno-Oncology Microbiome
